# TIPE2 suppresses angiogenesis and non-small cell lung cancer (NSCLC) invasiveness via inhibiting Rac1 activation and VEGF expression

**DOI:** 10.18632/oncotarget.11406

**Published:** 2016-08-19

**Authors:** Zequn Li, Chun Guo, Xianglan Liu, Chengjun Zhou, Faliang Zhu, Xiaoyan Wang, Qun Wang, Yongyu Shi, Jianing Wang, Wei Zhao, Lining Zhang

**Affiliations:** ^1^ Department of Immunology, Shandong University School of Medicine, Jinan, China; ^2^ Department of Pathology, The Second Hospital of Shandong University, Jinan, China

**Keywords:** TIPE2, NSCLC, angiogenesis, invasiveness, Rac1

## Abstract

Non-small cell lung cancer (NSCLC) is one of the leading causes of all cancer-related deaths worldwide. Despite extensive efforts to improve the diagnosis and treatment of this neoplasm, limited progress has been made. Tumor necrosis factor (TNF)-alpha-induced protein 8-like 2 (TIPE2 or TNFAIP8L2) is a newly introduced negative immune regulator, which also controls tumorigenesis. However, the role of TIPE2 in angiogenesis is unknown. In the present study, we investigated the expression and roles of TIPE2 in NSCLC. TIPE2 upregulation in human NSCLC tissues was negatively associated with the primary tumor size, lymph node metastasis, and advanced clinical stage, which can be used to predict lymph node metastasis. Moreover, overexpression of TIPE2 not only inhibited the colony formation, migration, and invasion of NSCLC cells but also indirectly suppressed the proliferation, migration, and tube formation of vascular endothelial cells. Furthermore, TIPE2 suppressed tumor invasiveness and angiogenesis via inhibiting the activation of Rac1 and subsequently weakening its downstream effects, including F-actin polymerization and VEGF expression. Collectively, these results indicate that TIPE2 plays a key role in NSCLC metastasis, suggesting that forced TIPE2 expression might be a novel strategy for the treatment of NSCLC.

## INTRODUCTION

Lung cancer is one of the most commonly diagnosed cancer and the leading causes of all cancer related deaths worldwide [1, 2]. The major histological types of lung cancer could be divided into squamous carcinoma, adenocarcinoma, large cell carcinoma, adeno-squamous carcinoma and small cell carcinoma. Except for small cell carcinoma, the other four types, for the convenience of clinical practice, are categorized as non-small cell lung cancer (NSCLC), which counts for about 75% to 80% of all lung cancer cases [3]. Generally lung cancer is treated with surgical resection combined with chemotherapy and certain kinds of targeted drugs. However, the long-term survival of NSCLC patients remains poor due to the high metastasis potential of the tumor cells [4]. It is known that enhanced invasiveness of tumor cells and formation of new blood vessels in tumor microenvironment are key steps during the process of tumor metastasis [5–7]. Therefore, discovering novel molecules that control tumor invasiveness and angiogenesis is of vital importance for improving the prognosis of NSCLC patients.

Tumor necrosis factor-α-induced protein-8 (TNFAIP8)-like-2 (TIPE2) belongs to TNFAIP8 family, which consists of four members, TNFAIP8, TIPE1, TIPE2 and TIPE3 [8]. It was initially discovered as an immune negative regulator and plays a vital role in regulating both innate and adaptive immunity via inhibiting Toll-like receptor (TLR) and T-cell receptor (TCR) activation pathway [8, 9]. It was first isolated from inflamed spinal cord of experimental autoimmune encephalomyelitis (EAE) mice and its deficiency leads to lethal multi-organ inflammation in 129 mice [9, 10]. As an immune regulator, TIPE2 is preferentially expressed in lymphoid tissues and is down-regulated in patients with infectious or autoimmune disorders [8, 9, 11]. What's more, human TIPE2 is also widely expressed in a variety of non-immune cells, suggesting that it may have other functions beyond immune regulation [12].

Recently, researches on TIPE2 and its related effects on tumor development have become a research focus. The expression conditions and effects of TIPE2 in some tumors were explored and discussed preliminarily [9, 13–17]. However, detailed mechanisms were limited in most of these researches, which needs to be further investigated. Our previous research demonstrated that by directly binding with Rac1-GTPases, murine TIPE2 dictates the strength of phagocytosis and oxidative burst in innate immunity, while human TIPE2 could inhibit the proliferation, migration and invasion *in vitro* and suppress the growth and metastasis of hepatocellular carcinoma (HCC) *in vivo* [18, 19]. Rac1 belongs to the Ras superfamily of small GTPases, which is involved in a variety of important cellular processes such as gene transcription, cell adhesion, cell movement and cell cycle progression [20, 21]. Targeting Rac1 and subsequently inhibiting its activity make TIPE2 a potential therapeutic strategy to suppress the invasiveness of tumor cells. The effect of TIPE2 on angiogenesis, another key step contributing to tumor metastasis, remains unclear till now.

In the present study, we demonstrated that TIPE2 was a promising biomarker to diagnose NSCLC and predict tumor metastasis. Moreover, TIPE2 suppressed tumor invasiveness and angiogenesis via inhibiting the activation of Rac1 and subsequently weakening its downstream effects, F-actin polymerization and VEGF expression. All these data indicate that TIPE2 may contribute to improving the diagnostic accuracy and therapeutic effect of NSCLC, which is deserved to be further explored.

## RESULTS

### TIPE2 protein expression was up-regulated in NSCLC tumor tissues compared with adjacent normal tissues

**Figure 1:**
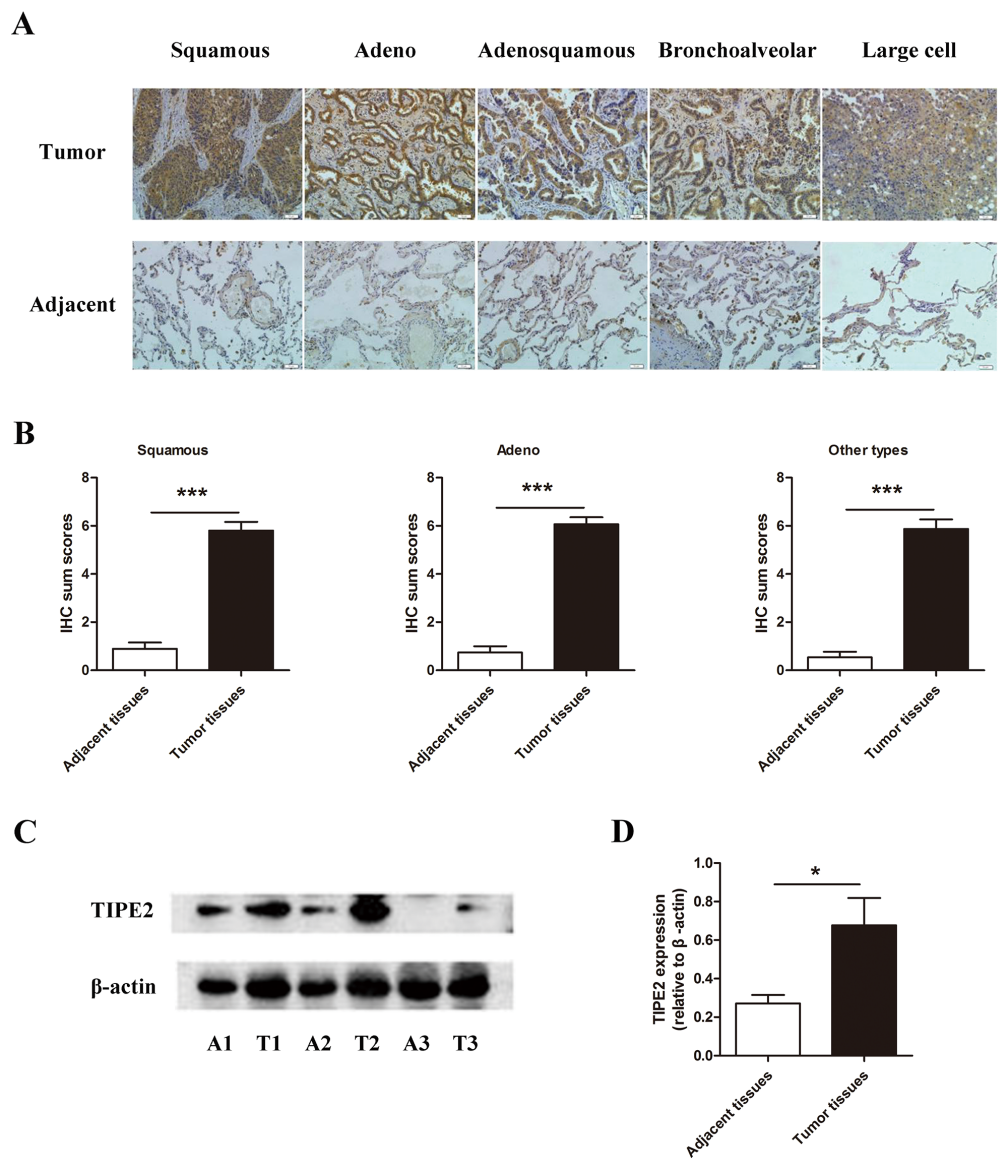
The expression of TIPE2 in NSCLC tissues **A.** IHC results (200×magnification) of TIPE2 expression in different subtypes of NSCLC tissues and adjacent tissues. **B.** IHC sum scores were used to compare TIPE2 expression in different subtypes of NSCLC tissues and adjacent tissues. **C.** Representative results of TIPE2 protein expression in fresh NSCLC tumor tissues (T) and adjacent normal tissues (A) detected by western blot. **D.** Statistical results showed that TIPE2 was significantly elevated in fresh NSCLC tissues compared to adjacent normal tissues. *, *P*<0.05; ***, *P*<0.001.

As NSCLC accounts for the majority of lung cancer, we focus on NSCLC in this study. To explore the expression of TIPE2 protein in NSCLC tissues, firstly we detected TIPE2 expression in NSCLC tissue chip that consists of 75 NSCLC specimens and corresponding adjacent tissues by immunohistochemistry (IHC). Results showed that comparing to adjacent tissues, TIPE2 protein was highly expressed in all histological subtypes of NSCLCs arrayed, including squamous carcinoma, adenocarcinoma, adeno-squamous carcinoma, bronchoalveolar carcinoma and large cell lung carcinoma (Figure 1A). As shown in Figure 1B and Table 1, statistical analysis showed that TIPE2 protein was significantly up-regulated in NSCLC tissues compared to normal tissues. Then we detected TIPE2 protein expression in 10 NSCLC fresh specimens, as well as the corresponding adjacent normal tissues (Figure 1C and 1D), the results further proved the aforementioned conclusions that TIPE2 expression was high in NSCLC tumor tissues and low in adjacent non-tumor tissues.

**Table 1:** TIPE2 expression in different subtypes of NSCLC tissues and corresponding adjacent nontumorous tissues

Histopathological classification	Number	TIPE2 expression		P value
		Low	High	
**Squamous carcinoma**				
Tumor tissues	30	8 (26.7%)	22 (73.3%)	<0.0001
Adjacent tissues		29(96.7%)	1(3.3%)	
**Adenocarcinoma**				
Tumor tissues	30	6 (20.0%)	24 (80.0%)	<0.0001
Adjacent tissues		28(93.3%)	2(6.7%)	
**Other types**				
Tumor tissues	15	4 (26.7%)	11(73.3%)	<0.0001
Adjacent tissues		15(100%)	0(0%)	

### TIPE2 expression was negatively associated with primary tumor size, lymph node metastasis and clinical stage in NSCLC

**Figure 2:**
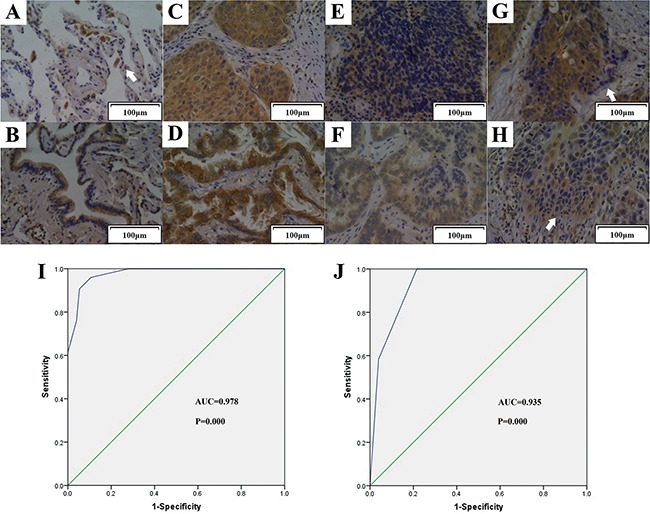
TIPE2 expression in normal lung tissue, tissue with metaplasia, NSCLC tissues with or without lymph node metastasis Representative figures (200×magnification) of TIPE2 expression in normal lung tissue A. (Arrow indicated macrophage), lung tissue with glandular metaplasia B. squamous cell carcinoma C. adenocarcinoma D. that without lymph node metastasis, squamous cell carcinoma E. adenocarcinoma F. that with lymph node metastasis, and tumor cells infiltrated into the stroma G. and H. (Arrows in G and H indicated tumor cells that infiltrated into the stroma) I. To test the diagnostic capability of TIPE2 expression in distinguishing cancerous tissues and noncancerous tissues, ROC curves was conducted and results manifested that strong separation between NSCLC tissues and corresponding adjacent noncancerous tissues, with an area under curve (AUC) of 0.978 (*P*=0.000). J. ROC curve was established and showed clear separation between patients with and without lymph node metastasis, with an AUC of 0.935 (*P*=0.000).

Results of IHC showed that TIPE2 expression was negative in the alveoli of normal lung tissues, but strong staining could be found in inflammatory cells such as plasmocytes and macrophages (Figure 2A). Previous research found that TIPE2 is preferentially expressed in squamous epithelium and glandular epithelium [22]. Consistent with these findings, increased TIPE2 expression was observed in lung tissues with glandular metaplasia (Figure 2B). More importantly, although TIPE2 was highly expressed in squamous cell carcinoma (Figure 2C) and adenocarcinoma (Figure 2D), we found that TIPE2 expression decreased markedly in tumor tissues with lymph node metastasis (Figure 2E and 2F). Moreover, TIPE2 staining was obviously weakened in the cells that infiltrated into the stroma (Figure 2G and 2H), indicating that TIPE2 may be associated with invasiveness and lymph node metastasis of NSCLC.

To determine the diagnostic value of TIPE2 expression in NSCLC, we constructed receiver operator characteristic (ROC) curves and calculated the area under the curve (AUC) to access whether TIPE2 expression was able to differentiate cancerous cases and noncancerous cases. The ROC curves showed that the AUC for TIPE2 in discriminating NSCLC cancerous cases and noncancerous cases was up to 0.978 (Figure 2I, CI (95%): 0.939-0.995, *P*=0.000) with high sensitivity, specificity, PPV (positive predictive value) and NPV (negative predictive value) (Supplementary Table S1).

To investigate the clinical significance of TIPE2 in NSCLC, we further analyzed the association of TIPE2 expression to clinicopathological parameters in NSCLC tissue chip (Table 2). Interestingly, TIPE2 expression was found to be negatively associated with primary tumor size (*P*=0.0159), clinical stage (*P*=0.0128) and lymph node metastasis (LNM) (*P*=0.0004), which is consistent with our aforementioned findings of IHC. To further explore whether TIPE2 could serve as a biomarker in predicting lymph node metastasis, ROC curve was constructed and AUC was calculated. Results showed that the AUC for TIPE2 in discriminating between cancerous tissues with lymph node metastasis and those without lymph node metastasis was up to 0.935 (Figure 2J, CI (95%): 0.854-0.979, *P*=0.000), the estimated sensitivity, specificity, PPV and NPV were 78.43%, 100%, 100% and 68.6%, respectively (Supplementary Table S2). All these data suggested that TIPE2 could serve as a promising biomarker for the diagnosis of NSCLC and prediction of tumor metastasis.

**Table 2:** Correlations between TIPE2 expression and clinicopathological characteristics in NSCLC tissues

Variable	Number	TIPE2 expression		P value
		Low	High	
**Age (years)**				
<60	33	11	22	0.1095
≥60	42	7	35	
**Gender**				
Male	47	11	36	1.0000
Female	28	7	21	
**Primary tumor size (cm)**				
<5	40	5	35	**0.0159**
≥5	35	13	22	
**Lymph node metastasis (LNM)**				
Negative (N0)	24	0	24	**0.0004**
Positive (N1-N3)	51	18	33	
**Histopathological classification**				
Squamous	30	8	22	0.8031
Adenoma	30	6	24	
Others	15	4	11	
**Clinical stage**				
I	11	0	11	**0.0128**
II	41	8	33	
III	23	10	13	

### TIPE2 overexpression markedly inhibited the colony formation, migration and invasion of NSCLC cells

**Figure 3:**
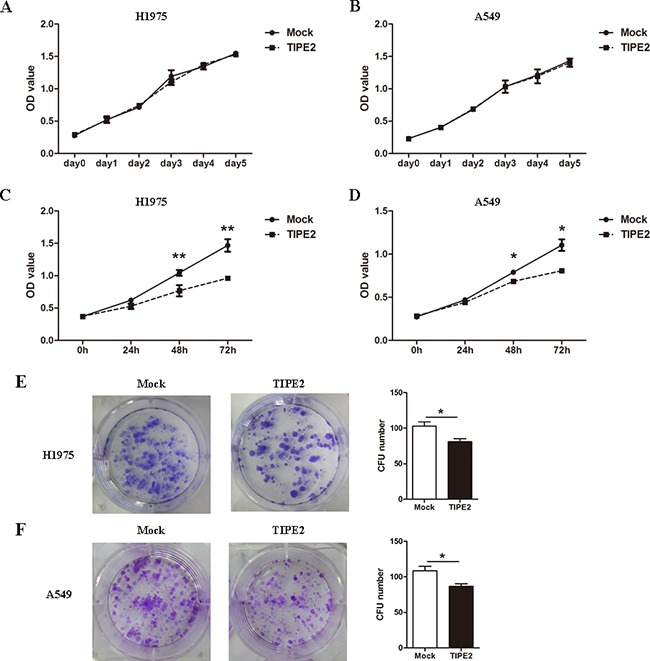
TIPE2 overexpression inhibited the colony formation but not the proliferation of NSCLC cells **A.** and **B.** After transfection of mock or wild type TIPE2 plasmid, CCK8 assays were conducted with a duration of 5 days. **C.** and **D.** After TIPE2 overexpression, TNF-α was used to stimulate the proliferation of tumor cells, then CCK8 assays were conducted at 24 hours, 48 hours and 72 hours after stimulation. **E.** and **F.** Two weeks after transient PRK5-TIPE2 plasmid transfection, colonies that consisted of more than 50 cells were counted. Data in (A) to (F) are mean±SD from three independent experiments with similar results. *, *P*<0.05; **, *P*<0.01.

To further elucidate the role of TIPE2 in NSCLC, we firstly examined the effects of TIPE2 on the proliferation of NSCLC cell lines, H1975 and A549, by CCK8 assays *in vitro*. PRK5-TIPE2 recombinant plasmid was constructed and transfected into H1975 cells and A549 cells. An empty plasmid (mock) was used as control. We found that TIPE2 overexpression in H1975 cells and A549 cells (Supplementary Figure S1A and S1B) had no effect on proliferation of the two cell lines within 5 days (Figure 3A and 3B). However, TIPE2 overexpression could suppress TNF-α induced cell proliferation in NSCLC cells (Figure 3C and 3D). Moreover, TIPE2 overexpression significantly attenuated the colony formation capability of both two cell lines (Figure 3E and 3F).

**Figure 4:**
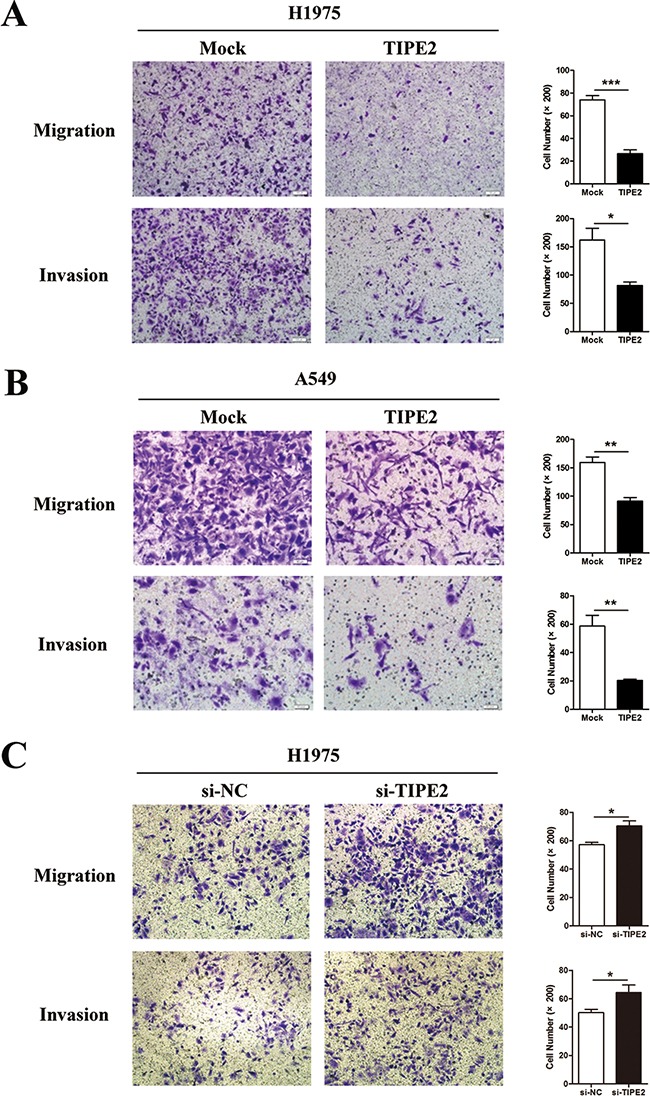
TIPE2 overexpression dramatically suppressed the migration and invasion of NSCLC cells **A.** to **B.** Transwell migration and invasion assays were conducted after TIPE2 overexpression in H1975 cells (A) and A549 cells (B); **C.** Transwell migration and invasion assays were conducted after transfection of TIPE2 siRNA in H1975 cells. Five fields of cells were counted in the lower chambers (200×magnification). Data represent mean±SD from three independent experiments. *, *P*<0.05; **, *P*<0.01; ***,*P*<0.001.

As the migration and invasion capacities of tumor cells are key steps during the process of tumor metastasis, we next assessed the effect of TIPE2 on migration and invasion in NSCLC cells. The results indicated that TIPE2 overexpression significantly reduced the migration and invasion capacities of H1975 cells (Figure 4A) and A549 cells (Figure 4B). To further elucidate the inhibitory effect of TIPE2 on migration and invasion, Transwell assays were conducted after TIPE2 siRNA transfection in H1975 cells, as TIPE2 expression in H1975 was higher than that in A549, and the latter cells showed nearly negative TIPE2 expression. TIPE2 expression was significantly decreased at both mRNA level and protein level after transfection with TIPE2 specific siRNA. (Supplementary Figure S1C and 1D). Compared with the control cells, TIPE2 siRNA markedly increased both the migration and invasion capacities of H1975 cells (Figure 4C). Taken together, these results indicated that TIPE2 had no impact on the proliferation of NSCLC cells but markedly inhibited their migration, invasion and colony formation.

### Tumor conditioned medium derived from TIPE2-overexpressed NSCLC cells suppressed the proliferation, migration and tube formation of vascular endothelial cells

**Figure 5:**
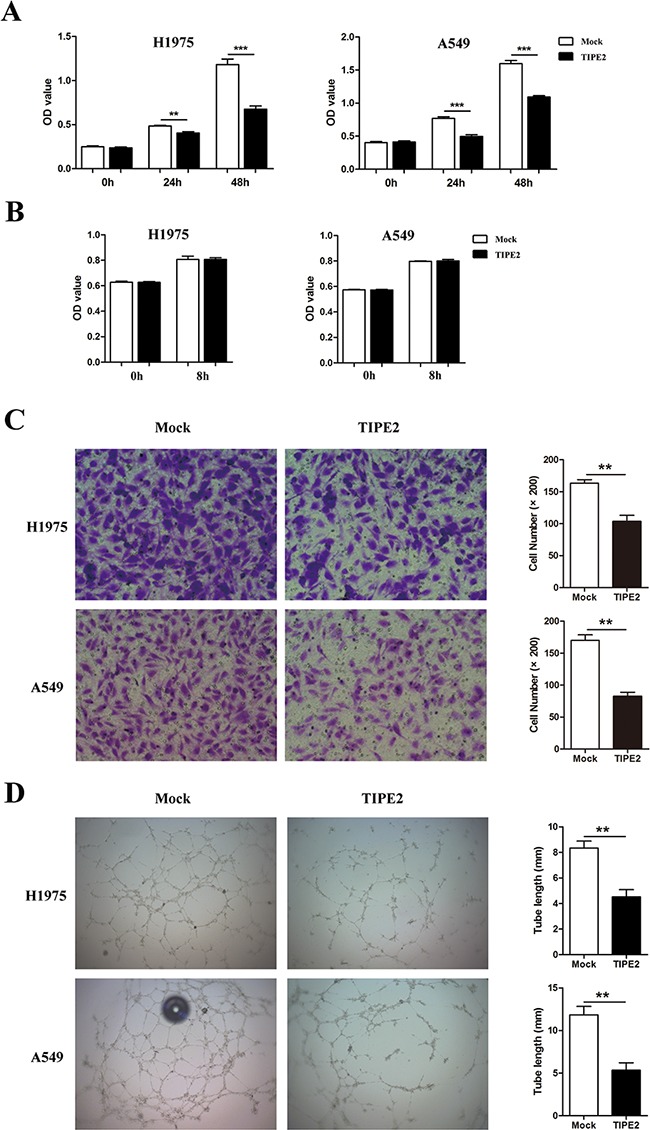
TIPE2 dramatically suppressed the proliferation, migration and tube formation of HUVECs **A.** HUVECs were cultured with TCMs derived from mock or TIPE2 transfected NSCLC cells. Cell viability was assessed by CCK8 assays at indicated time points. **B.** Cell viability of HUVECs that cultured with TCMs was detected by CCK8 assays at 0h and 8h. **C.** HUVECs were cultured with TCMs derived from mock or TIPE2 transfected NSCLC cells. Transwell assays were conducted to analyze the effect of TIPE2 on the migration capacity of HUVECs. Five fields of cells in the lower surface were counted (200×magnification). **D.** HUVECs cultured with TCMs derived from mock or TIPE2 transfected NSCLC cells were used for Matrigel tube formation assay. Data represent mean±SD from three independent experiments. **, *P*<0.01; ***, *P*<0.001.

An important feature of tumor progression is the achievement of adequate vascular development to provide nutrition and environment for hematogenous metastasis [23]. So we further analyzed the role of TIPE2 expression in tumor cells on angiogenesis. Human umbilical vein endothelial cells (HUVECs) were cultured in different tumor conditioned medium (TCM) derived from NSCLC cells transfected with mock plasmid or TIPE2 plasmid and then the proliferation, migration as well as tube formation of OHUVECs were analyzed. We found that TCM derived from TIPE2-overexpressed NSCLC cells could significantly suppress the viability of HUVECs (Figure 5A). Further, we investigated the influence of TIPE2 TCM on the migration capacity of HUVECs. To exclude the impact of proliferative difference on cell migration, we tested whether TIPE2 could affect cell viability within 8 hours, which was the exact time needed for the migration assay. Results revealed that after 8 hours of TCM treatment, no significant difference in cell viability was shown between TIPE2 TCM and mock TCM treated cells (Figure 5B). Moreover, we found that TIPE2 TCM significantly inhibited the migration of HUVECs (Figure 5C). In addition, results showed that formed capillary structures of HUVECs treated by TIPE2 TCM were significantly decreased (Figure 5D). All these results revealed that TCM derived from TIPE2-overexpressed NSCLC cells obviously suppressed the proliferation, migration and tube formation of vascular endothelial cells.

### TIPE2 suppressed the colony formation, migration and invasion of NSCLC cells and the angiogenesis of HUVECs by inhibiting the activity of Rac1

**Figure 6:**
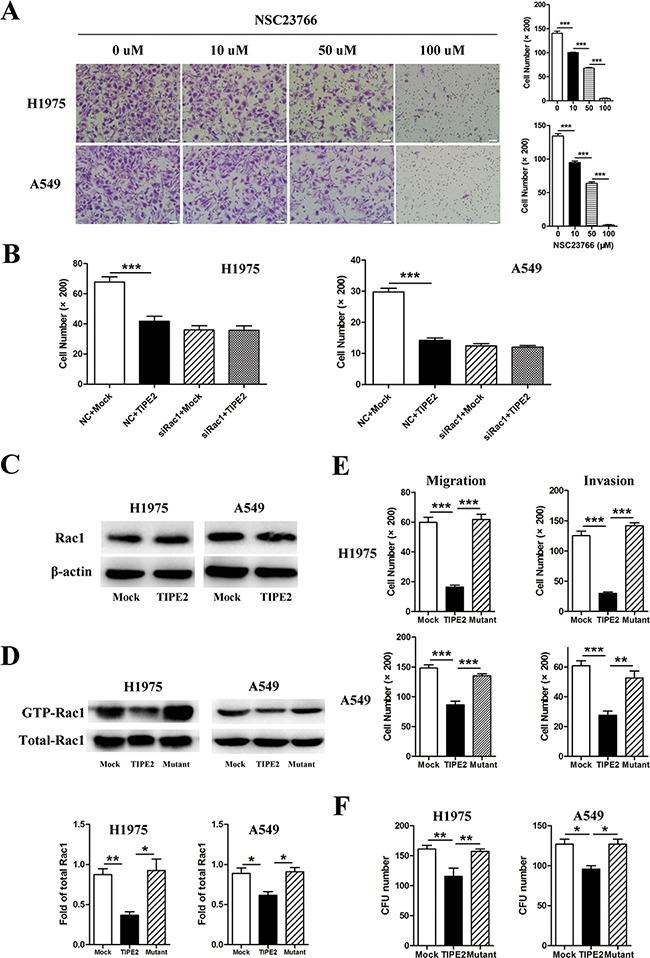
TIPE2 suppressed the colony formation, migration and invasion of NSCLC cells via inhibiting Rac1 pathway **A.** H1975 and A549 cells treated with different concentration of NSC23766, a Rac1 inhibitor, were used for migration assays. **B.** H1975 and A549 cells co-transfected with Rac1 specific siRNA and mock or TIPE2 plasmids were used for migration assays. **C.** Total Rac1 expression in H1975 and A549 after transfected with mock and wild type TIPE2 plasmids was detected by western blot. **D.** After transfected with mock, wild type TIPE2 and mutant TIPE2 plasmids respectively, cell lysates of H1975 and A549 were used for PAK-GST pull down assays. Activated Rac1 that pulled down as well as total Rac1 in the lysates were detected by western blot analysis. **E.** H1975 and A549 cells that transfected with mock, wild type TIPE2 and mutant TIPE2 plasmids were used for migration and invasion assays. **F.** H1975 and A549 cells that transfected with mock, wild type TIPE2 and mutant TIPE2 plasmids were used for colony formation assays. Data are mean±SD and the results shown were representative of 3 independent experiments. Five fields of cells in the lower compartment were counted in Transwell assays (200×magnification). *, *P*<0.05; **, *P*<0.01; ***, *P*<0.001.

As the aforementioned results indicated, TIPE2 was able to suppress the colony formation, migration and invasion of NSCLC cells, we further explored the possible mechanism. Some researches demonstrated that Rac1 is involved in the metastasis and angiogenesis of lung cancer [24–26]. Moreover, our previous researches indicated that TIPE2 could inhibit Rac1 activation in innate immune cells and in human hepatocellular carcinoma (HCC) [18, 27]. So we speculated that TIPE2 may suppress the metastasis and angiogenesis by inhibiting Rac1 activity in NSCLC. To confirm our hypothesis, we performed the following experiments. Firstly, we detected the expression of Rac1 and its effect on the migration capacities of NSCLC cells. We found that both H1975 and A549 expressed high level Rac1 and NSC23766, a specific Rac1 activity inhibitor, effectively suppressed the migration capacity of NSCLC cells in a dose dependent manner (Figure 6A and 6C). Furthermore, silencing of Rac1 expression by specific siRNA eliminated the inhibitory effect of TIPE2 on the migration and invasion of NSCLC cells (Figure 6B). These results indicated that TIPE2 may function through the Rac1 pathway. Thirdly, we detected the impact of TIPE2 on expression of total Rac1 by western blot and Rac1 activation by GST pull-down assay. As shown in Figure 6C and 6D, TIPE2 had no effect on total amount of Rac1 expression (Figure 6C) but significantly decreased Rac1-GTPase expression. More importantly, the mutation of TIPE2 in sites which binds to Rac1 [19], reversed this inhibitory effect (Figure 6D). Moreover, mutation of TIPE2 also reversed its inhibitory effect on the migration, invasion (Figure 6E), colony formation (Figure 6F) of H1975 and A549 cells. All these results demonstrated that TIPE2 suppressed the colony formation, migration and invasion of NSCLC cells via inhibiting Rac1 activity.

**Figure 7:**
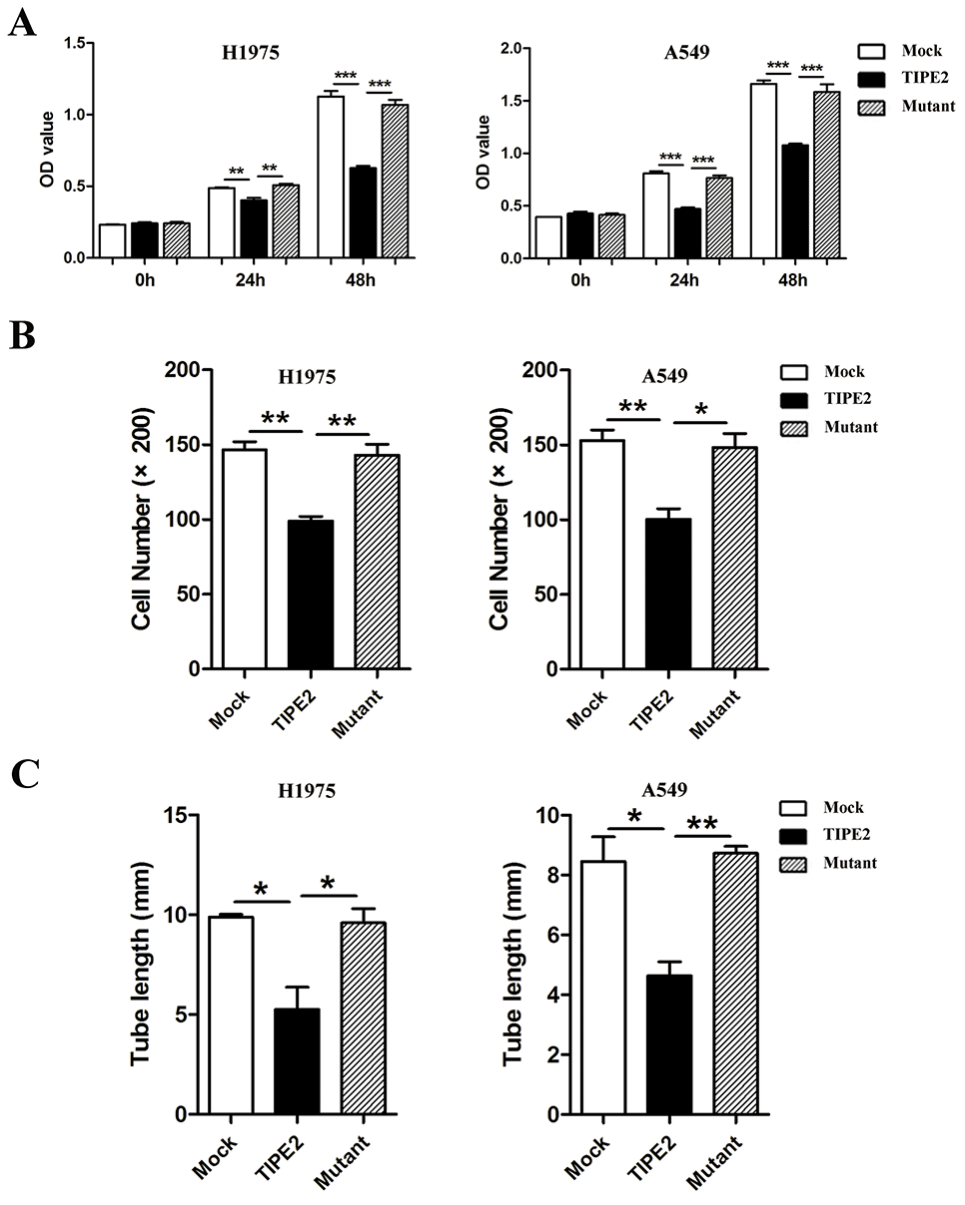
TIPE2 suppressed the proliferation, migration and tube formation of HUVECs by inhibiting Rac1 pathway Different kinds of TCMs derived from NSCLC cells that transfected with mock, wild type TIPE2 and mutant TIPE2 plasmids were used for CCK8 assays **A.** Transwell migration assays **B.** and tube formation assays **C.** in HUVECs. Data represent mean±SD from three independent experiments. Five fields of cells in the lower compartment were counted in Transwell assays (200×magnification). *, *P*<0.05; **, *P*<0.01; ***, *P*<0.001.

Furthermore, to detect whether Rac1 is also required for TIPE2 TCM-mediated inhibition of angiogenesis, we examined the proliferation, migration and tube formation capacity of HUVECs treated with TCM derived from mock, wild type TIPE2, and mutant TIPE2 plasmid transfected cancer cells, respectively. Results showed that the inhibitory effects of wild type TIPE2 TCM on the proliferation (Figure 7A), migration (Figure 7B) and tube formation (Figure 7C) of HUVECs were reversed by TIPE2 mutation, indicating that TIPE2 TCM suppressed the angiogenesis of HUVECs via inhibiting Rac1 activity.

### TIPE2 suppressed Rac1 downstream effectors, F-actin polymerization and VEGF expression in NSCLC cells

**Figure 8:**
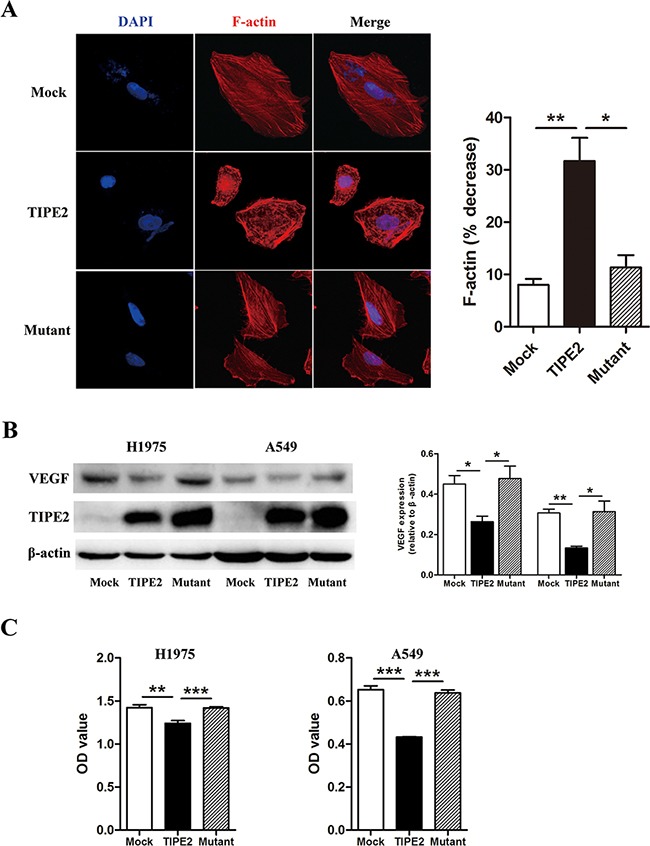
TIPE2 decreased F-actin polymerization, VEGF expression and VEGF-A secretion via inhibiting Rac1 pathway **A.** After transfected with mock, wild type TIPE2 and mutant TIPE2 plasmids respectively, F-actin in H1975 cells was stained by TRITC-conjugated phalloidin and observed with laser confocal microscopy. The “% decrease in F-actin”=[(F-actin in untreated cells–F-actin in mock, wild type TIPE2 or mutant plasmids treated cells)/F-actin in untreated cells]×100. **B.** The expression of VEGF was detected in H1975 cells and A549 cells by western blot after transfected with mock, wild type TIPE2 and mutant TIPE2 plasmids respectively. **C.** The expression of VEGF-A that secreted into the supernatant of H1975 and A549 cells, which were transfected with mock, wild type TIPE2 and mutant TIPE2 plasmids previously, were detected by VEGF-A ELISA kit. Data are mean±SD and the results shown were representative of 3 independent experiments. *, *P*<0.05; **, *P*<0.01; ***, *P*<0.001.

It has been well accepted that microfilaments play a key role in cell motility, thus influencing the migration and invasion of tumor cells. Rac1 functions as an engine to provide energy for microfilaments dynamics [28]. Therefore, we explored the effect of TIPE2 on the organization of microfilaments in H1975 cells. We found that there was a significant microfilament depolymerization after TIPE2 overexpression. Moreover, this effect was reversed by TIPE2 mutation (Figure 8A), indicating that TIPE2 suppressed microfilament polymerization via inhibiting Rac1. VEGF is critical for angiogenesis and also appears to be an essential factor for the progression of many tumors. Among its subtypes, VEGF-A is mostly studied, which could promote the proliferation, migration and tube formation of HUVECs [29]. As shown in Figure 8B and 8C, TIPE2 significantly decreased the expression of VEGF in H1975 and A549 cells, as well as secreted VEGF-A in the supernatant of these cells. What's more, this effect vanished when mutant TIPE2 plasmid was used. In addition, our previous research found that TIPE2 suppressed MMP9, uPA expression by inhibiting Rac1 in HCC cells [18]. However, no significant changes of MMP9 or uPA expression was shown in NSCLC cells after TIPE2 overexpression (Supplementary Figure S2). Taken together, TIPE2 suppressed the invasiveness of NSCLC cells and the angiogenesis via inhibiting Rac1 dependent Factin polymerization and VEGF expression.

## DISCUSSION

Tumor metastasis is the major reason of death in patients with NSCLC and novel targeted treatment approaches are required. In the present study, we found that TIPE2 suppresses tumor invasion and angiogenesis in a Rac1 dependent manner and it also serves as a novel biomarker for prediction of tumor metastasis, suggesting forced TIPE2 expression might be a novel strategy for the treatment of NSCLC.

Limited data suggest that status of TIPE2 expression in tumors may be tissue-specific. TIPE2 expression is lower in tumor tissues of HCC [18] and gastric cancer [17] than that in adjacent non-tumor tissues, while on the contrary, its expression is higher in tumor tissue of colon cancer [16] and renal cell carcinoma [30] compared with that in normal tissues. One report showed that TIPE2 expression was significantly higher in normal lung tissues compared with NSCLC [31] and other reports revealed that TIPE2 expression was significantly decreased in lung squamous cancer and small cell lung cancer tissues compared to the non-tumor lung tissues, while there existed no difference between lung adenocarcinoma and non-tumor lung tissues [32]. Our previous research has shown that TIPE2 expression is higher in normal squamous epithelium and bronchial columnar epithelial cells but is low or absent in alveolar epithelial cells [22]. In line with that in normal lung tissue, here we found that TIPE2 expression was also lower in alveolar epithelial cells but markedly higher in bronchial columnar epithelial cells and macrophagocytes in alveolar lumen in adjacent non-tumor lung tissues. The increased expression of TIPE2 in NSCLC tumor tissues made us doubt that TIPE2 may promote the development of NSCLC as that in colon cancer, different from that in HCC, where it acts as a tumor suppressor. However, further analysis revealed that TIPE2 expression decreased with tumor progression and its reduced expression in tumor tissues was significantly associated with large primary tumor size and more lymph node metastasis. ROC analysis indicated that TIPE2 expression also had discriminative validity in distinguishing lymph node metastasis. Furthermore, forced TIPE2 expression could markedly inhibit the migration, invasion and colony formation of NSCLC cells while silencing of TIPE2 promoted these effects *in vitro*. Therefore, our data indicate that although TIPE2 expression was obviously high in tumor tissues than in adjacent non-tumor lung tissues, TIPE2 still serves as a tumor suppressor in NSCLC, which is supported by the recent two reports [31, 32]. In addition, our data also suggested that TIPE2 may play suppressive role in advanced stage of NSCLC since TIPE2 expression was high in early stage of NSCLC tumor tissues, similar to normal columnar epithelial cell or squamous epithelium, while it was low in advanced stage with lymph node metastasis. Taken together, we deduced that TIPE2 expression was up-regulated with the process of metaplasia and tumorigenesis, and with the development of tumor, the expression of TIPE2 decreased, accompanied with the enhanced malignant behaviors of tumor cells.

Of note, we demonstrated for the first time that TIPE2 not only directly inhibited the colony formation ability, migration and invasion of NSCLC cells but also indirectly suppressed the proliferation, migration and tube formation of vascular endothelial cells via inhibiting VEGF expression and secretion by NSCLC cells. It is well accepted that angiogenesis is one of the key steps during the process of tumor metastasis as it provides nutrition and way for hematogenous metastasis [5, 27, 33]. We found that TCMs derived from TIPE2 transfected NSCLC cells showed decreased VEGF expression and markedly inhibited the proliferation, migration and tube formation of HUVECs. By suppressing angiogenesis, the volume of tumors with high TIPE2 expression may be limited due to that enough nutrition was not available, which may account for the phenomenon that decreased TIPE2 expression was associated with larger primary tumor size.

The mechanism by which TIPE2 inhibits tumor remains complicated, depends on a variety of factors. Liu et al. reported that TIPE2 may promote apoptosis of lung cancer by regulating expression of apoptotic molecules, increasing expression of caspase-3, caspase-9 and Bax, and decreasing expression of Bcl-2 [32]. Zhao et al. showed that TIPE2 may inhibit gastric cancer cell proliferation via activating p27 [17]. Our previous research indicated that human TIPE2 suppressed the migration and invasion of HCC cells via inhibiting Rac1 pathway [18]. Rac1 is a member of the Rho family of small GTPases and has been implicated in a wide variety of cellular processes, including cytoskeletal reorganization and gene transcription [34]. The overexpression of small GTPases is correlated with poor prognosis in NSCLC [35]. Besides promoting cell motility and cytoskeleton polymerization [36, 37], Rac1 activation is also of vital importance in the process of angiogenesis via promoting VEGF expression and blood vessel lumen morphogenesis. VEGF secreted by tumor cells accelerates the process of angiogenesis thus promotes hematogenous metastasis [38, 39]. VEGF is so critical for tumor development that a variety of targeted drugs have already been applied for clinical practice and improved the prognosis of patients significantly [40, 41]. Research found that Rac1 is involved in the metastasis progress and drug resistance in NSCLC [42, 43]. All the aforementioned data indicate that Rac1 is an ideal target for preventing tumor metastasis. Therefore, targeting at Rac1 may be considered as a promising therapeutic approach for the treatment of advanced lung cancer [26, 33, 44]. In this study, by the use of specific Rac1 inhibitor, Rac1 siRNA and mutant TIPE2 plasmid, we demonstrated that TIPE2 suppressed the migration, invasion, colony formation capacities and the angiogenesis of NSCLC via blocking Rac1 downstream signals. Rac1-GTPase is known to promote lamellipodia formation at the leading edge of migrating cells, which are involved in cancer metastasis [45], this may accounts for the phenomenon that TIPE2 expression was decreased markedly in tumor cells that infiltrated into the stroma. Interestingly, unlike that TIPE2 suppresses MMP9 and uPA expression in HCC [18], TIPE2 had no effect on MMP9 and uPA expression in NSCLC, this may be explained by tumor specificity.

In conclusion, TIPE2 is an endogenous inhibitor of Rac1 in NSCLC. By inhibiting Rac1 activity and suppressing the corresponding downstream effects, such as F-actin polymerization and VEGF expression, TIPE2 functions as a tumor suppressor to control the migration, invasion of NSCLC cells and the angiogenesis in tumor microenvironment. The decreased TIPE2 expression in NSCLC is a promising biomarker for predicting tumor lymph node metastasis. Forced TIPE2 expression may be considered as a potential therapeutic strategy in controlling the metastasis of NSCLC.

## MATERIALS AND METHODS

### Tissue samples

A total of 75 non-small cell lung cancer specimens (including 30 squamous cell carcinoma specimens, 30 adenocarcinoma specimens, 7 adenosquamous carcinoma specimens, 5 bronchoalveolar carcinoma specimens and 3 large cell carcinoma specimens) and the corresponding adjacent normal tissues from NSCLC tissue chip (OUTDO BIOTECH, Shanghai, China) were used for the detection of TIPE2 expression by IHC. Other 10 specimens were collected from patients who underwent surgery at Qilu Hospital of Shandong University, which were used for TIPE2 detection by western blot. Clinical and clinicopathological classification and staging were determined according to the American Joint Committee on Cancer (AJCC) criteria [46]. The study was approved by the Institutional Review Board of Shandong University, China and informed consent was obtained from each subject.

### Cell culture

The human NSCLC cell lines, H1975 and A549, obtained from the American Type Culture Collection (ATCC), were separately maintained in RPMI 1640 and DMEM medium (Gibco, CA, USA) supplemented with 10% inactivated fetal bovine serum (FBS) (Gibco) in a humidified cell incubator with an atmosphere of 5% CO2 at 37°C.

### Plasmid construction and transfection

The wild type TIPE2 was generated from the cDNA clone by PCR and cloned in frame with a C-terminal Flag into vector PRK5. The mutant TIPE2 in which the TIPE2 N-terminal lysine or arginine residues, Lys-15, Lys-16, and Arg-24 were replaced with glutamine or alanine was generated by PCR-based site-directed mutagenesis as previously described [19]. The TIPE2-siRNA sequences purchased from GenePharma were as follows: forward 5′-GCACAUUCCACCUUGACAATT-3′; reverse 5′-UUGUCAAGGUGGAAUGUGCTT-3′. The negative control siRNA sequence: forward 5′-UUCUCCGAACG UGCUACGUTT-3′, reverse 5′-ACGUGACACGUUCGG AGAATT-3′. Specific siRNA for Rac1 (forward 5′-GCA AACAGAUGUGUUCUUA-3′, reverse 5′-UAAGAAC ACAUCUGUUUGC-3′) and nonspecific negative control (forward 5′-UUCUCCGAACGUGUCACGUTT-3′, reverse 5′-ACGUGACACGUUCGGAGAATT-3′) were purchased from Sigma-Aldrich (Louis, USA). Transfection of NSCLC cells with plasmid or siRNA was performed using Lipofectamine 2000 according to the manufacturer's protocols (Invitrogen, Carlsbad, CA, USA).

### RNA isolation, RT-PCR and real-time quantitative PCR

Total RNAs were extracted from transfected cells using TRIzol reagent (Invitrogen, Carlsbad, CA, USA) and were reverse transcribed into cDNA using a Rever-Tra Ace qPCR Kit (Toyobo, Osaka, Japan). RT-PCR was performed using 2×Taq MasterMix (CWBIO, Beijing, China). Real-time PCR was performed using an UltraSYBR Mixture (CWBIO). The sequences of the sense and antisense primers were as follows: TIPE2: 5′-ACTGA GTAAGATGGCGGGTCG-3′, and 5-TTCTGGCGAA AGCGGGTAG-3′; Rac1: 5′-AT GTCCGTGCAAAGTGGTATC-3′, and 5-CTCGGATCGCT TCGTCAAACA-3′; GAPDH: 5′-AACGGATTTGGTCGT ATTGGG-3′, and 5′-CCTGGA AGATGGTGATGGGAT-3′; MMP9: 5-GCATTCAGGGAGACGCCCATTT AACGA CA-3′, and 5′-CTGACACTCCCGGTGGG AAATCA-3′; uPA: 5′-ACTACATTGTCTACCTGGGTCGGTC-3′ and 5′-ATGCAA GATGAGTTGCTCCACTTC-3′. Relative gene expression levels were normalized to GAPDH as control.

### Western blot

The protein extract was dissolved in a cell lysis buffer containing 1% protease inhibitor. Protein concentration of the homogenized lysates was measured using a BCA method (Sangon, Shanghai, China). Equal amount of protein was separated by SDS-PAGE and then transferred to PVDF membranes (Millipore, Billerica, MA, USA). Membranes were probed overnight at 4°C with the following primary antibodies: rabbit polyclonal antibody against human TIPE2 (1:300; BOSTER, Wuhan, China), rabbit monoclonal antibody against VEGF (1:2000; Millipore, CA, USA), the matrix metalloproteinase 9 (MMP-9) and urokinase-PA (u-PA) (1:1000; EPITOMICS, Hangzhou, China), mouse monoclonal antibody against Rac1 (1:300; Abcam, Hongkong), β-actin (1:1000; ZSGB-Bio, Beijing, China), followed by secondary antibodies (1:2000; goat anti rabbit or mouse IgG, ZSGB-Bio) conjugated with peroxidase for 1 h at room temperature. After washing, signals were visualized by eECL Western Blot Kit (CWBIO).

### Immunohistochemistry (IHC)

Immunohistochemistry was performed using NSCLC tissue chip. The tissue chip was dewaxed and hydrated, followed by antigen retrieval (in 0.01mol/L citrate buffer solution, pH6.0, heated to boil for 2-3 min in a stainless steel pressure cooker). Endogenous peroxidase was blocked using a 3% hydrogen peroxide solution. The section was incubated with the blocking goat serum for 15 min, then immunostained with rabbit antibody against TIPE2 (dilution 1:200) at 4^°^C overnight. Secondary staining was performed with HRP-conjugated anti-rabbit IgG using a MaxVsion Kit and a 3, 5-diaminobenzidine (DAB) peroxidase substrate kit (Maixin Co, Fuzhou, China). The tissue chip was counterstained with hematoxylin. For negative controls, the primary antibody was replaced with PBS.

### Evaluation of immunohistochemical staining

The immunohistochemical staining was independently evaluated by two experienced pathologists in a blinded manner. Staining was semi-quantitatively scored based on both the staining intensity (0, negative; 1, weak; 2, moderate; 3, strong) and the percentage of positively stained cells (0, 0%; 1, 1%-25%; 2, 26%-50%; 3, 51%-75%; 4, 76%-100%). The two scores for each specimen were then combined to come up with a final TIPE2 expression score. The cut-off point of the sum of the scores were defined as follows: 0-3, low expression; 4-7, high expression. The appropriateness of the cut-off point was validated by ROC analysis.

### Colony formation assays

Cells were seeded in six-well plates at a density of 500 cells per well and every 3 days the medium was replaced. After 2 weeks, cells were washed by PBS and fixed with methanol for 10 minutes, then stained with 1% crystal violet. Colonies that consisted of more than 50 cells were counted and calculated as a percentage of that to the control group. The experiment was independently performed for at least three times.

### Cell viability assays

A total of 3000 cells were seeded in 96-well plates with triplicate wells and cultured for indicated time points. Cell viability was evaluated using CCK8 (Beyotime, Haimen, China) assay according to manufacturer's instructions. The absorbance was determined at 450 nm wave length. Each time point was repeated in three wells and the experiment was independently performed at least for three times.

### Transwell assays for cell migration and invasion

The migration and invasion capacities of tumor cells were analyzed in 24-well Boyden chambers with 8-μm pore size polycarbonate membranes (Costar, Acton, USA). For invasion assay, the membranes were precoated with 50 μg Matrigel (BD Biosciences, San Diego, USA) to simulate matrix barriers. Cells (5 × 105/ml) were resuspended in 200 μl serum-free medium and placed in the upper chamber. The lower compartments were filled with 600 μl medium with 10% FBS. After incubation (incubation time for migration is 10-12 hours and for invasion is 24 hours), the cells remaining on the upper surface of the membrane were removed. The cells on the lower surface of the membrane were fixed with methanol for 10 minutes and then stained with crystal violet for 20 minutes. Stained cell counting was performed under a light microscope at ×200 magnification. NSC23766 (Calbiochem, San Diego, USA), a specific Rac1 inhibitor, was used to inhibit Rac1 activity in some experiments.

### Immunofluorescence (IF) for F-actin staining

In order to observe the effect of TIPE2 on F-actin, NSCLC cells were seeded (1× 104) on the cover slip and allowed to adhere overnight. Twenty-four hours after transfection, cells were fixed, permeabilized and then stained with Tetramethylrhodamine (TRITC)-conjugated phalloidin (Sigma–Aldrich, Louis, USA) for 1 hour. Nuclei were stained with 4′, 6-diamidino-2-phenylindole (DAPI) (Beyotime) for 5 min. The results were analyzed with confocal laser microscopy (Carl Zeiss, LSM780, Oberkochen, Germany). The “% decrease in F-actin”=[(F-actin in untreated cells–F-actin in mock, wild type TIPE2 or mutant plasmids treated cells)/F-actin in untreated cells]×100.

### Rac1 activity assay

The levels of the active GTP-bound form of Rac1 were determined with p21-activated kinase (PAK)-GST protein beads (Cytoskeleton). Cell lysate was prepared as previously described [19]. The lysate was incubated with 20 μg of PAK-GST protein beads for 30 min at 4°C. After washing, proteins on beads and in total cell lysates were assessed by SDS-PAGE followed by immunoblotting with a Rac1-antibody.

### Preparation of tumor conditioned medium (TCM)

NSCLC cells were seeded into 6-well plate and transfected with different plasmids, 24 hours after transfection, the supernatants were collected and centrifuged at 1000 rpm for 10 minutes to eliminate cell masses, then centrifuge the supernatants at 12000 rpm for 10 minutes to get rid of cell debris. The supernatant was collected as TCM for further study.

### VEGF-A ELISA assay

The levels of soluble VEGF-A in TCMs was detected using a VEGF-A ELISA Kit (R&D Systems, Minneapolis, MN, USA) according to the manufacturer's instruction [47].

### Matrigel tube formation assays *in vitro*

HUVECs were cultured in TCM from the supernatant of NSCLC cells at a density of 15000 cells per well in a 96-well plate precoated with 50 μl thick Matrigel. The HUVECs were then maintained at 37°C in a 5% CO2 atmosphere for 6 hours. Capillary tube formation was calculated under a light microscope at ×40 magnification. Tube length was measured by Image-Pro Plus software (Media Cybernetics, L.P, Silver Spring, MD, USA) and was expressed as a total length (mm) per field for each well.

### Statistical analysis

All statistical analyses were carried out using SPSS 18.0 software (SPSS Inc., Chicago, USA). The chi-square test was used to analyze the relationship between TIPE2 expression and clinicopathological parameters. Receiver operating characteristics (ROC) curve analysis was performed to assess the diagnostic value of TIPE2 in NSCLC. Student's t test was used for comparison among different groups. P < 0.05 was considered to be statistically significant.

## SUPPLEMENTARY FIGURES AND TABLES


