# Supplementary material for: TIPE2 suppresses angiogenesis and non-small cell lung cancer (NSCLC) invasiveness via inhibiting Rac1 activation and VEGF expression
Source: Oncotarget. 2016 Aug 19;7(38):62224–39. doi: 10.18632/oncotarget.11406 (PMC5308722; doi:10.18632/oncotarget.11406)
Supplement: Supplementary file 1 [file oncotarget-07-62224-s001.pdf]

TIPE2 suppresses angiogenesis and non-small cell lung cancer (NSCLC) invasiveness via inhibiting Rac1 activation and VEGF expression

SUPPLEMENTARY FIGURES AND TABLES

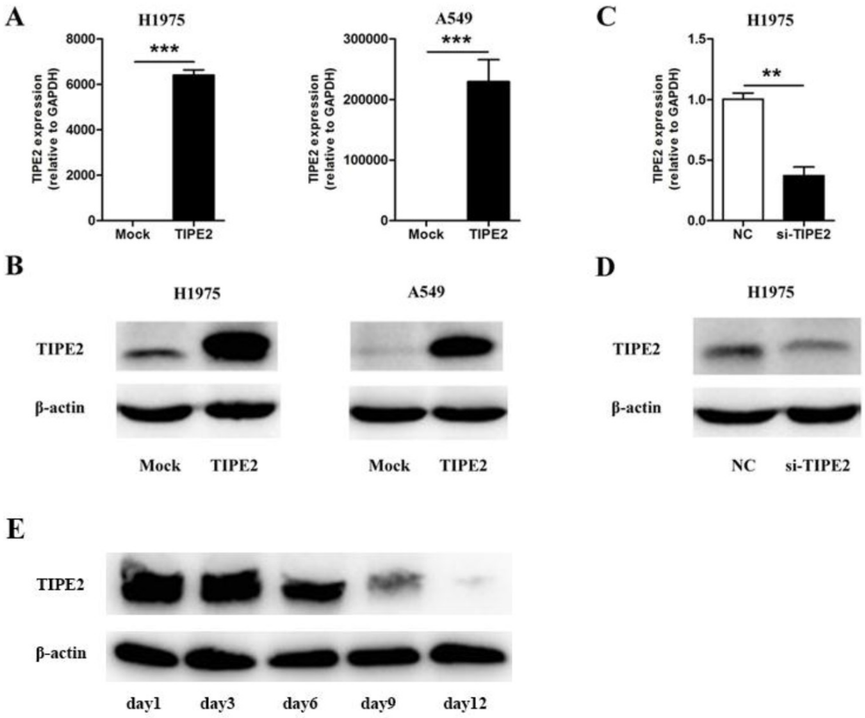

Supplementary Figure S1: Effects of transfection that validated by real-time PCR and western blot. A. TIPE2 mRNA expression in H1975 and A549 was detected by real-time PCR after transient transfection with mock or TIPE2 plasmid. **B.** TIPE2 protein expression in H1975 and A549 was detected by western blot after transient transfection with mock or TIPE2 plasmid. **C.** TIPE2 mRNA expression in H1975 cells was detected by real-time PCR after transient transfection with TIPE2 siRNA or negative control. **D.** TIPE2 protein expression in H1975 cells was detected by western blot after transient transfection with TIPE2 siRNA or negative control. **E.** TIPE2 protein expression in H1975 cells was detected by western blot 1, 3, 6, 9, 12 days after transient transfection with TIPE2 plasmid respectively. Data represent mean±SD from three independent experiments. *, $P<0.05$; **, $P<0.01$; ***, $P<0.001$.

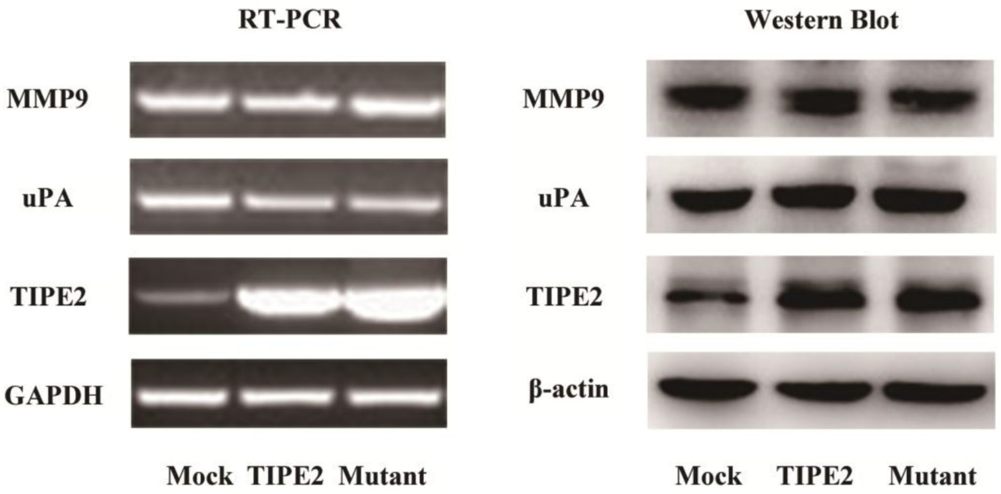

Supplementary Figure S2: TIPE2 had no effect on the expression of neither MMP9 nor uPA in H1975 cells. The expression of MMP9 and uPA were detected by RT-PCR and western blot in H1975 cells after transient transfection with mock, wild type TIPE2 and mutant TIPE2 plasmids respectively.

Supplementary Table S1: sensitivity, specificity, and positive and negative predictive values for NSCLC detection using TIPE2 expression

TIPE2 expression	Tumor	Adjacent	Sensitivity	Specificity	PPV[a]	NPV[b]
Low	18	72	90.67	94.67	94.4	91.0
High	57	3				

[a]PPV= Positive Predictive Value
[b]NPV= Negative Predictive Value

Supplementary Table S2: sensitivity, specificity, and positive and negative predictive values for LNM detection using TIPE2 expression

TIPE2 expression	LNM[a] negative	LNM positive	Sensitivity	Specificity	PPV[b]	NPV[c]
Low	0	18	78.43	100	100	68.6
High	24	33				

[a]LNM= Lymph node metastasis
[b]PPV= Positive Predictive Value
[c]NPV= Negative Predictive Value
